# Genomic selection for target traits in the Australian lentil breeding program

**DOI:** 10.3389/fpls.2023.1284781

**Published:** 2024-01-03

**Authors:** Alem Gebremedhin, Yongjun Li, Arun S. K. Shunmugam, Shimna Sudheesh, Hossein Valipour-Kahrood, Matthew J. Hayden, Garry M. Rosewarne, Sukhjiwan Kaur

**Affiliations:** ^1^ Agriculture Victoria, AgriBio, Centre for AgriBioscience, Bundoora, VIC, Australia; ^2^ Agriculture Victoria, Grains Innovation Park, Horsham, VIC, Australia; ^3^ School of Applied Systems Biology, La Trobe University, Bundoora, VIC, Australia

**Keywords:** BayesR, multi-environmental trial, genetic gain, genomic selection, lentil

## Abstract

Genomic selection (GS) uses associations between markers and phenotypes to predict the breeding values of individuals. It can be applied early in the breeding cycle to reduce the cross-to-cross generation interval and thereby increase genetic gain per unit of time. The development of cost-effective, high-throughput genotyping platforms has revolutionized plant breeding programs by enabling the implementation of GS at the scale required to achieve impact. As a result, GS is becoming routine in plant breeding, even in minor crops such as pulses. Here we examined 2,081 breeding lines from Agriculture Victoria’s national lentil breeding program for a range of target traits including grain yield, ascochyta blight resistance, botrytis grey mould resistance, salinity and boron stress tolerance, 100-grain weight, seed size index and protein content. A broad range of narrow-sense heritabilities was observed across these traits (0.24-0.66). Genomic prediction models were developed based on 64,781 genome-wide SNPs using Bayesian methodology and genomic estimated breeding values (GEBVs) were calculated. Forward cross-validation was applied to examine the prediction accuracy of GS for these targeted traits. The accuracy of GEBVs was consistently higher (0.34-0.83) than BLUP estimated breeding values (EBVs) (0.22-0.54), indicating a higher expected rate of genetic gain with GS. GS-led parental selection using early generation breeding materials also resulted in higher genetic gain compared to BLUP-based selection performed using later generation breeding lines. Our results show that implementing GS in lentil breeding will fast track the development of high-yielding cultivars with increased resistance to biotic and abiotic stresses, as well as improved seed quality traits.

## 1 Introduction

Lentil is a self-pollinating, diploid (2n = 14), cool season legume crop grown in temperate climates, with world production increased from 3.15 to 6.54 million metric tons in the last two decades (Kaale et al., 2023). Canada is a major producer of lentils, contributing to more than 50% to global trade, while Australia makes up another 10-15% (Sadras et al., 2021). In 2020-21, Australia produced 900,000 tons of lentil of which 864,403 tons were exported, accounting for 13.3% of global trade (ABARES, 2021; Semmler, 2021). Over the past 3 decades, the National Australian lentil breeding program achieved an average annual genetic gain of 1.2% using conventional breeding and management practices (Sadras et al., 2021). These findings align with global trends in lentil productivity over the past six decades, as reported by Kumar et al. (2021). Despite this good rate of genetic gain, it is below the level required to meet increasing global demand caused by population growth, as well as the changing dietary habits in western countries, where people are opting for nutritious, sustainable, and healthier foods. Lentil grain has about 26% protein content, which makes it an attractive choice for plant-based diets (Ismail et al., 2020). To keep up with this ever-increasing demand, it is critical to increase and stabilize lentil crop production through the: 1) development of varieties with high yield potential; 2) cropping area expansion; and 3) continuous germplasm improvement to withstand changing climatic conditions.

Recent studies have demonstrated considerable yield variations in lentil cultivation across diverse environments in Australia, with an average production of 1.2 tons/ha (Sadras et al., 2021; Suri et al., 2022). These variations arise primarily from the influence of various biotic and environmental stress factors. Of these, ascochyta blight, botrytis grey mould, boron and salinity have been identified as key limiting factors across a wide range of production regions (Davidson et al., 2016; Sudheesh et al., 2016; Rodda et al., 2017; Rodda et al., 2018; Dissanayake et al., 2020; Kumar et al., 2021) that can result in yield losses of 30-40% (Kumar et al., 2013). Climate variability and complex genotype-by-environment (G × E) interactions on the expression of phenotypic traits also contribute to low genetic gain in lentil breeding (Kumar and Ali, 2006; Kumar et al., 2020). As more than 95% of the Australian lentil crop is exported, grain quality traits such as grain weight, seed size and protein content are also important in meeting market demands. The abovementioned traits, as well as crop yield improvement, remain the primary focus of lentil breeding in Australia.

The advent of DNA markers opened new opportunities in plant breeding that have enabled breeders to make more informed and accurate selections through marker-assisted selection (MAS) (Kaur et al., 2014; Jain et al., 2017). In lentils, quantitative trait loci have been identified for ascochyta blight resistance, boron and salinity toxicity tolerance, yield, winter hardiness, seed weight, seed size and milling quality (Taylor et al., 2006; Barrios et al., 2007; Kaur et al., 2014; Verma et al., 2015; Sudheesh et al., 2016; Singh et al., 2017; Subedi et al., 2018; Singh et al., 2020). While some success has been achieved in the use of MAS to accelerate genetic gain in breeding programs, it is neither effective nor practical for quantitative traits, which are controlled by multiple genes with minor effects (Galli et al., 2020).

GS uses genome-wide marker information to estimate the genetic potential of an individual and has emerged as a dominant approach for genetic improvement in plant and livestock breeding, where selections are made based on genomic estimated breeding values (GEBVs) (Meuwissen et al., 2001). GS involves developing genomic prediction equations using genotyping and phenotyping data obtained from a training population, which are then used to estimate the GEBVs of individuals in a testing population that have not been phenotyped. GS is more beneficial for traits with low heritability and that are difficult to measure. It can outperform conventional phenotypic and MAS in terms of genetic gain per unit time and cost (Jannink et al., 2010). The primary advantage of GS is the ability to predict the phenotypic performance of individuals earlier in the breeding cycle, which reduces the cross-to-cross generation interval and increases genetic gain (Lin et al., 2021). GS was shown to provide a three-fold and two-fold increase in genetic gain in maize and wheat, respectively, compared to MAS (Heffner et al., 2010). The adoption of GS in maize breeding has reduced overall breeding costs by 30-50% (Crossa et al., 2017; Beyene et al., 2019). Other studies using simulated data have shown GS is superior to phenotypic selection, both in terms of shortening the breeding interval and increasing genetic gain per unit of time (Gaynor et al., 2017; Gorjanc et al., 2018; Li et al., 2022). Previous studies on lentils have also revealed that GS can increase genetic gain for economically important agronomic traits by reducing both cost and breeding cycle time (Haile et al., 2020; Janghel and Sharma, 2022; Li et al., 2022). Additionally, GS has been also studied in other grain legumes, such as common bean, field pea, and chickpea, as evidenced by several published studies (Roorkiwal et al., 2018; Annicchiarico et al., 2019; Keller et al., 2020; Diaz et al., 2021). However, it is worth noting that these studies have limited discussions on the practical implications of implementing GS in crop breeding.

Another advantage of GS is that it can handle G×E interactions effectively, allowing breeders to select germplasm across multiple environments. Several studies have assessed the impact of incorporating G×E into genomic prediction models and reported an increase in prediction accuracy. For example, a multi-environment study in barley showed an increase in prediction accuracy from 0.37 to 0.45 when G×E was included in the prediction model (Lin et al., 2021). Other studies have shown that the incorporation of environmental covariates and crop models into genomic prediction models can increase prediction accuracy by up to 11% in both tested and untested environments (Heslot et al., 2014; Jarquín et al., 2017). More recently, (Jighly et al., 2021) described an extension to existing GS models that incorporates genotype plus genotype-environment (GGE) analysis, which provided a 70% increase in prediction accuracy compared to GS models that only included G×E.

In the current study, we utilised historical phenotyping data collected across 10 years (2010-2020) for key target traits from Agriculture Victoria’s national lentil breeding program (NLBP). The objectives of this study were to: 1) develop genomic prediction models for target traits and calculate GEBVs to make selections, and 2) compare the expected rate of change in genetic gain per unit of time using GS and BLUP-based phenotypic selections.

## 2 Materials and methods

A total of 2,081 lentil genotypes including advanced breeding lines – Stage 2 (F_4_:F_7,_ 1,496 genotypes) and Stage 3 (F_4_:F_8_, 569 genotypes) and 16 commercial cultivars sourced from the NLBP were used as a training population (**Table 1**). Phenotyping data for nine traits were sourced including: grain yield (GYD), ascochyta blight (AB) resistance (two pathovars; one associated with lentil variety PBA Hurricane XT (ABH), and another one with variety Nipper (ABN)), boron (BOR) tolerance, salinity (SAL) tolerance, botrytis grey mould (BGM) resistance, 100-grain weight (GWT), seed size index (SSI) and protein content (PRO). GYD and BGM were evaluated under field conditions whereas ABN, ABH, BOR and SAL were assessed under controlled environment conditions. Grain quality traits PRO, SSI and GWT were measured in a seed phenomics laboratory. All the phenotyping experiments included the use of released cultivars as checks and controls. A total of 241 individual experiments from 16 locations were analysed in this study. **Table 1** summarises the traits investigated in this study.

**Table 1:** A descriptive summary of the total number of observations and trials for each trait assessed in the current study.

Trait	No. of observations	No. of trials	Mean	SD
**GYD**	48,336	132	1.80	0.96
**ABH**	1,772	5	7.61	9.63
**ABN**	1,920	5	2.36	5.01
**BOR**	3,988	17	44.58	19.91
**SAL**	6,659	22	5.14	2.01
**BGM**	8,913	27	5.79	1.60
**GWT**	3,234	11	4.05	0.60
**SSI**	3,234	11	4.46	0.25
**PRO**	3,234	11	26.74	1.71

GYD (tons/ha); BOR (1-100, percentage necrosis); SAL (Score 1-10); BGM (Score 1-9); ABH, (percentage area of plant disease); ABN (percentage area of plant disease); GWT (g); SSI (index); PRO (percentage).

### 2.1 Trait phenotyping

#### 2.1.1 Grain yield

GYD was evaluated in 132 field trials across 16 locations over 20 growing seasons between 2010 and 2020 in four Australian states: South Australia (SA), Victoria (VIC), New South Wales (NSW) and Western Australia (WA) (**Supplementary Table 1**). These trials followed a randomised complete block design with released varieties as checks (at least five checks per trial) and were managed under rainfed conditions. Stage 2 trials were replicated twice, whilst stage 3 trials were replicated three times. GYD was expressed in tons/ha and extrapolated from the plot harvest. Each plot size was 1.25 m (wide) × 5 m (long) (6.25 m² area) with 0.25 m spacing between rows. A summary of the GYD trials is provided in **Supplementary Table 2**.

#### 2.1.2 Ascochyta blight resistance screening

AB screening was performed as described in (Davidson et al., 2016; Sudheesh et al., 2016) with minor modifications using two different *Ascochyta lentis* isolates (virulent to PBA Hurricane XT and Nipper lentil varieties) (**Supplementary Table 3**). Seeds of each lentil line were seeded into two replications per line, with two to three seeds per pot, and then placed in plastic tents (160×80×80 cm) in a controlled environment room (CER) at 15°C, 12/12-h light/dark. Trials were set up in a randomised complete block design, with one replication per tent and susceptible and resistant check plants included in the set. Experiments used a randomised complete block design, with one replicate per tent with susceptible and resistant checks included. Seedlings were inoculated two weeks after sowing and disease symptoms on each seedling were assessed and visually scored 10-14 days later as percentage area of plant disease (% APD).

#### 2.1.3 Boron tolerance screening

BOR toxicity tolerance was assessed using a hydroponic screening method as described in (Rodda et al., 2018)), where six plants per genotype were grown in trays of peat plugs floating in a dilute boric acid solution for two weeks. Lentil varieties previously characterized as intolerant (PBA Blitz and Cassab) and tolerant (ILL2024) were used as internal controls to determine the ideal assessment time. The genotypes were scored based on percentage necrosis using a 0-100 scale, where low score values indicated tolerance and high score values indicated susceptibility to boron toxicity.

#### 2.1.4 Salt tolerance screening

A pot-based salinity tolerance screening method was used as described in (Dissanayake et al., 2020). In brief, the study included 1,755 lines with each experiment conducted as a randomized complete block design with two replications and six plants per pot. Salt was applied in the form of diluted commercial nutrient solution (Nitrosol®, Amgrow Pty. Ltd., Lidcombe, New South Wales, Australia (nitrogen:phosphorous:potassium (NPK) 4:1:3) and Ca(NO_3_)_2_H_2_O at 20% of the recommended concentration) in increments of 2 ds/m per day to reach the desired level of 6 ds/m. Each plant’s response to salinity stress was assessed 10 weeks post-sowing using the visual growth response scale (1-10) developed by Maher and Connor (2003). The average growth response scale values for each pot were used for analysis.

#### 2.1.5 Botrytis grey mould tolerance screening

BGM resistance was assessed in the field under natural infection conditions that occurred in GYD field trials (stages 2 and 3) over two years (four locations in 2013 and seven locations in 2016). The Horsham, Melton, Mallala, and Williamulka experiments were scored in 2013, and Beulah, Curyo, Kadina, Mallala, Melton, Rupanyup, and Wagga Wagga were scored in 2016. Each experiment used a randomised complete block design with two and three replications for stages 2 and 3 genotypes, respectively. Disease symptoms were assessed on individual plots using a 1-9 scoring scale, where 1 indicated no infection and 9 indicated over 50% of plants within a plot were infected.

#### 2.1.6 Grain quality traits screening

Grain quality traits were assessed in the seed phenomics laboratory, Horsham, Victoria. SSI was obtained using the EyeFoss™ (FOSS Analytical, Hoganas, Sweden) image analysis as described in LeMasurier et al. (2014). Near-infrared spectroscopy (NIRS) was used to determine PRO, whereas GWT was calculated as described by (Revilla et al., 2019).

### 2.2 Genotyping and SNP data calling

All stage 2 and 3 materials (2010-2020) were genotyped using the imputation enabled multispecies pulse 30K SNP array, containing 10,528 lentil specific SNPs. In brief, DNA extractions were performed from 6 seeds per sample using a modified CTAB protocol (Tibbits et al., 2008). A total of 200 ng DNA per sample was used for the genotyping assay following the manufacturer’s protocols for the Infinium XT SNP bead chip array (Illumina Inc., San Diego, USA). Initial analysis was performed using GenomeStudio 2.0 Polyploid software (Illumina) using the manufacturer’s supplied crop-specific SNP manifest file. Theta and normalized R values were exported from GenomeStudio and used to call SNP using the custom genotype calling pipeline. Phasing and filling of missing data was performed using Eagle/Beagle (Browning and Browning, 2007; Browning and Browning, 2016). Imputation to the whole genome sequence level (3,528,788 SNPs) was achieved using Minimac3 (Das et al., 2016) and whole genome sequence (WGS)-based reference haplotypes. The imputed SNP data was filtered for linkage disequilibrium (r^2^ > 0.99) with a window size of 250kb, minor allelic frequency (MAF<0.05), heterozygosity per SNP (>20%) and heterozygosity per sample (>50%) using vcftools (Danecek et al., 2011) and bcftools (Li, 2011). A final set of 64,781 filtered SNPs were used for GS. Due to the usage of proprietary breeding material in this study, the genotype and marker names have been de-identified in the genotyping data file (Supplementary text file 1). However, the distribution of SNP markers along the lentil genome is shown in **Supplementary Figure 1**.

### 2.3 Grain yield clustering

Pairwise genetic correlations calculated between different experiments across multiple environments were found to be quite low. Consequently, as an alternative approach to include G×E in the analysis, six environmental variables (daily evaporation (mm), solar radiation (MJ/m^2^), rainfall (mm), vapour pressure (KPa), and minimum and maximum temperatures (degree in Celsius)) were used to cluster the GYD trials. Data for the environmental variables corresponding to each of the 132 GYD trials was obtained from the Scientific Information for Land Owners (SILO) (https://www.longpaddock.qld.gov.au/silo/) for the duration of lentil growing season (May- early December; 2010-2020), with the nearest meteorological station selected. The number of clusters was then determined by applying hierarchical clustering to the environmental variable data. For hierarchical clustering, dissimilarity values between clusters were computed using the Euclidean distance (*d_E_*) in (Equation 1), (Fox and Rosielle, 1982).


(1) $$d_{E}\left(i,j\right)=\sqrt{\sum_{k=1}^{6}(X_{ik}-X_{jk)}{}^{2}}$$

where *X_ik_* is the value for environmental variable *k* for cluster *i*,*X_jk_* is the value for environmental variable *k* for cluster *j* where *i* and *j* = 1,…,4, and k = 1,…,6. We applied the hclust function in R to implement clustering by the distance matrix (Smoliński et al., 2002).

### 2.4 Statistical analyses

#### 2.4.1 Spatial adjustment

The broad-sense heritability (*H^2^*) of traits measured from each trial was estimated using the linear mixed model shown in Equation 2.


(2) y=Xb+Zg+e

where *y* is the vector of phenotypic observations, *b* is a vector of fixed effects (the population mean and replicates), and *g* is a vector of random total genetic effects with a normally-distributed variance structure $N\left(0,\sigma_{g}^{2}I\right)$ , where $\sigma_{g}^{2}\;$ is the total genetic variance and *I* is the identity matrix; and *e* is a vector of residuals with a variance matrix *R*. *X* and *Z* are incidence matrices that link phenotypic observations to the fixed and random effects, respectively. The residual variance matrix R is decomposed into spatially dependent ( ξ ) and spatially independent (η) residuals by fitting autoregression (AR) of rows and columns with the formula below in Equation 3 (Dutkowski et al., 2006):


(3) $$R=\sigma_{\xi }^{2}\left[AR\left(\rho_{c}\right)⊗AR\left(\rho_{r}\right)\right]+\sigma_{\text{η}}^{2}I,$$

where $\sigma_{\xi }^{2}$ is the spatially dependent residual variance, $\sigma_{\text{η}}^{2}$ is the spatially independent residual variance, $\rho_{c}$ and $\rho_{r}$ are the autoregression parameters on column and row, respectively.

The broad-sense heritability was calculated as $H^{2}=\frac{\sigma_{g}^{2}}{\sigma_{g}^{2}+{\text{ση}}_{2}}$ . For genomic analyses, the best linear unbiased estimator (BLUE) of individuals tested within a trial was estimated using (Equation 2) by fitting individuals as fixed effects and no random effects.

#### 2.4.2 Genomic selection analyses

The vector of BLUEs of measured traits were modelled with a general form of the mixed linear models as shown in Equation 4:


(4) y=Xb+Za+e

where *b* is a vector of fixed effects (the population mean), *a* is a vector of the additive genetic effects and *e* is a vector of the residual effects. *X* and *Z* are incidence matrices that link phenotypic observations to the fixed and random effects, respectively. For genomic selection, the marker effects were estimated first in the training population and then genomic breeding values of the validation population were calculated. The marker effects were estimated using a linear mixed model shown in Equation 4 and implemented with BayesR package developed by (Breen et al., 2022).

The vector of the additive genetic effects *a* are the SNP effects in BayesR with an incidence matrix *Z* with $z_{ij}=\;\frac{z_{ij}^{*}-2p_{j}}{\sqrt{2p_{j}\left(1-p_{j}\right)}}$ , where $z_{ij}^{*}$ is the genotype of individual *i* at SNP *j*. The SNP effects were modelled by a mixture of four categories of distributions as defined by (Breen et al., 2022)): one for SNPs with zero effect, one for very small effects, one for small to medium effects and one medium to large, with a cumulative genetic variance $\sigma_{\text{a}}^{2}\;$ explained by SNPs. *e* is a vector of residual effects following $e\sim N\left(0,\sigma_{e}^{2}I\right)$ , $\sigma_{e}^{2}$ is the residual variance. The BayesR was implemented using a Markov Chain Monte Carlo (MCMC) process with 50,000 iterations and 25,000 burn-in in 5 chains. Narrow-sense heritability was calculated as $h^{2}=\frac{\sigma_{a}^{2}}{\sigma_{a}^{2}+{\text{σe}}_{2}}$ . Genomic estimated breeding values ( $\hat{g}$ , GEBVs) were predicted as the linear combination of marker effects as under (Equation 5):


(5) $$\hat{g}=Xa$$

where *X* is an incidence matrix of SNP genotypes and *a* is a vector of the SNP effects that were estimated from Equation 4. Prediction accuracy of the GEBVs was estimated as the Pearson correlation between GEBVs and BLUEs.

#### 2.4.3 Genetic correlation for GYD between clusters

Pair-wise genetic correlations between GYD clusters were estimated using ASReml-R (Butler et al., 2009). The genetic correlation between a pair of clusters was calculated as $r_{g}=\frac{\sigma_{a_{\text{ij}}}}{\sqrt{\sigma_{a_{\text{i}}}^{2}*\sigma_{a_{\text{j}}}^{2}}}$ . In Equation 4, the random additive genetic effects *a* have a normal distribution following a normal distribution $a\sim N\left(0,G_{A}⊗G\right)$ , where $G_{A}=\left[\begin{array}{cc} \sigma_{a_{\text{i}}}^{2} & \sigma_{a_{\text{ij}}}\\ \sigma_{a_{\text{ji}}} & \sigma_{a_{\text{j}}}^{2} \end{array}\right]$ , where $\sigma_{a_{i}}^{2}$ is the additive genetic variance for cluster *i*, $\sigma_{a_{j}}^{2}$ is the additive genetic variance for cluster *j*, $\sigma_{a_{ij}}$ is the additive genetic covariance between cluster *i* and cluster *j*, *G* is the genomic relationship matrix (GRM) estimated from SNP genotyped using VanRaden et al. (2009), ⊗ denotes the Kronecker product. The residual effects e follow a normal distribution e~N(0,R⊗I), , where $R=\left[\begin{array}{cc} \sigma_{e_{\text{i}}}^{2} & 0\\ 0 & \sigma_{e_{\text{j}}}^{2} \end{array}\right]$ , where $\sigma_{e_{i}}^{2}$ is the residual variance for cluster *i*, $\sigma_{e_{j}}^{2}$ is the residual variance for cluster *j*.

#### 2.4.4 Investigation of genomic prediction accuracy

Three validation scenarios were used to investigate the prediction accuracy of genomic breeding values: five-fold, Leave One year Out Validation (LOOV) and forward validation. In the five-fold validation, individuals were equally divided into five groups randomly, and the GEBVs of one group were predicted using training data from the remaining four groups. In LOOV, the GEBVs of one year were predicted using data of the remaining years for model training. For forward validation, the GEBVs of current years were predicted using data of previous years for model training. For all scenarios, the genotypes that overlapped with the training were removed from the validation to prevent overestimating the prediction accuracy.

Within-cluster and across-cluster validation were used to develop prediction accuracies for GYD. All three validation scenarios – namely five-fold, LOOV, and forward validation – were applied for prediction accuracy within-clusters. Furthermore, all validation scenarios were applied to the entire population (without clustering on the GYD data) in a non-G×E validation method. For the across-cluster validation data, validation was initially carried out between clusters, with one cluster being predicted using data from all other clusters as a training set. Secondly, validation was performed between clusters by predicting one cluster using data from another cluster. For all other target traits, non-G×E interactions were used in all validation scenarios across the whole population.

#### 2.4.5 Efficiency of GS over BLUP selection

To evaluate the efficiency of GS over BLUP-based selection, the best linear unbiased prediction (BLUP) EBVs of traits examined were estimated based on BLUEs and pedigree information using the model specified in Equation 4 and implemented with ASReml-R (Butler et al., 2009). The random additive genetic effects *a* follow a normal distribution with $Var\left(a\right)\sim N\left(0,\sigma_{a}^{2}A\right)$ , where $\sigma_{\text{a}}^{2}$ is the additive genetic variance and *A* is the numerator relationship matrix calculated from pedigree (Mrode, 2014). Forward validation was conducted based on BLUP EBVs to derive the accuracy of BLUP EBVs for the comparison between GS and BLUP selection.

Expected genetic gain of GS over BLUP selection based on EBVs was calculated for different scenarios of selecting parents from different stages of a breeding cycle: Stage 2, F_6_, F_2_ and F_1_. The generation intervals were chosen as defined by Li et al. (2022): 8.5, 5, 1 and 0.5 years for selecting parents from Stage 2, F_6_, F_2_ and F_1_ in GS and 8 years for BLUP selection. Additional expected genetic gain obtained from GS over BLUP selection was calculated in Equation 7 as that described by (Li et al., 2019):


(7) $$E=\frac{i\cdot \frac{r_{IH_{g}}}{L_{g}}\cdot \sigma_{A}-i\cdot \frac{r_{IH_{a}}}{L_{a}}\cdot \sigma_{A}}{i\cdot \frac{r_{IH_{a}}}{L_{a}}\cdot \sigma_{A}}=\frac{\frac{r_{IH_{g}}}{L_{g}}-\frac{r_{IH_{a}}}{L_{a}}}{\frac{r_{IH_{a}}}{L_{a}}}$$

where *i* is the selection intensity, $r_{IH_{g}}$ is the accuracy of GS estimated from the forward validation, $r_{IH_{a}}$ is the accuracy of BLUP selection estimated from the forward validation based on EBV, $\sigma_{A}\;$ is the square root of the additive genetic variance, *L_g_* is the generation interval of lentil breeding population under GS and *L_a_* is the generation interval of lentil breeding population under BLUP selection.

## 3 Results

### 3.1 Clustering of grain yield

**Figure 1:**
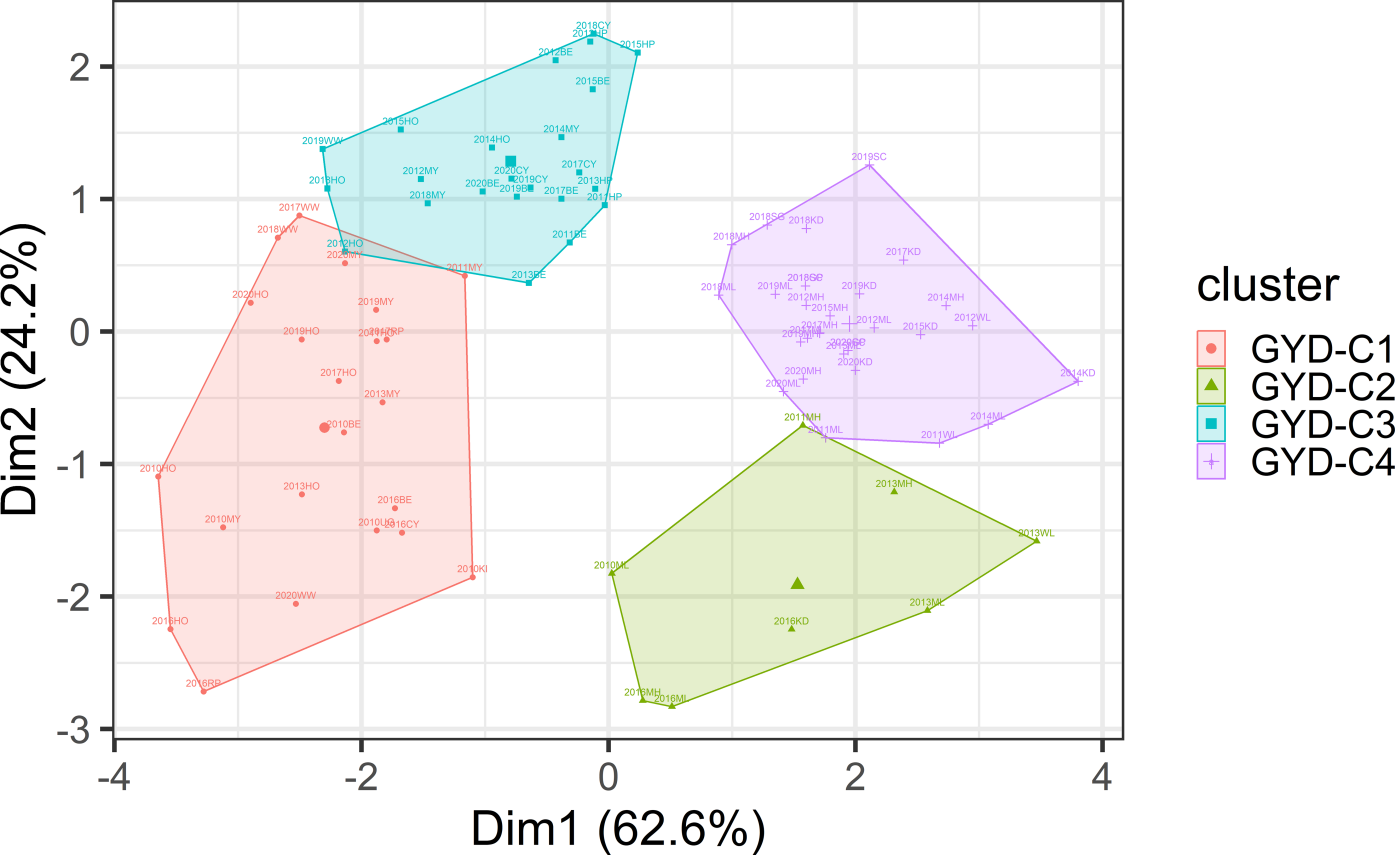
Clustering of GYD data based on environmental variables of daily evaporation, solar radiation, rainfall, vapour pressure, and temperatures (Min and Max).

Based on six environmental variables, the 132 yield trials were grouped into four clusters using hierarchical clustering (**Figure 1**). Most of the variation (86.8%) was explained by Dimensions 1 and 2. Total rainfall and evaporation were found to be the most important climatic factors. GYD-C1 was marked by high rainfall and low evaporation, GYD-C2 with high rainfall and high evaporation, GYD-C3 with low rainfall and low evaporation, and GYD-C4 with low rainfall and high evaporation. The number of trials grouped into GYD-C1, GYD-C3 and GYD-C4 was 33, 38 and 47 which were screened across 8, 9 and 8 years, respectively (**Table 2**). Only 14 trials from 4 years were grouped into GYD-C2.

**Table 2:** Summary of environmental variables for trial sites within each grain yield cluster, as well as all trials sites for grain yield.

Cluster	MTE	MDSR	MTR	MDVP	MDMT	MDXT	NoY
GYD†	721.0	13.5	246.6	10.6	6.6	19.2	11
GYD-C1	561.4	12.6	326.9	10.4	5.7	18.0	8
GYD-C2	819.9	14.2	347.2	11.5	8.0	19.4	4
GYD-C3	664.0	13.4	188.6	10.0	5.7	19.3	9
GYD-C4	859.9	14.1	203.8	11.0	7.7	20.1	8

† Data in all clusters was combined, ignoring G×E interactions on GYD, MTE, mean total evaporation; MDSR, mean daily solar radiation; MTR, mean total rainfall; MDVP, mean daily vapour pressure; MDMT, mean daily minimum temperature; MDXT, mean daily maximum temperature; NoY, number of years when trials within cluster were evaluated.

### 3.2 Phenotypic and genotypic variations of target traits

The average mean BLUE for GYD (tons/ha) was 1.8, with GYD-C1 and GYD-C2 having the highest and GYD-C3 having the lowest yield (**Table 3**). For all other traits, spatial adjustment was performed for each individual trial. We also evaluated connectedness (number of genotypes in common between environment), where the trials shared genotypes ranging from 3 to 341 (**Supplementary Table 4**). For most traits, moderate to high levels of pairwise genetic correlations were observed among different environments over years, except (>=0.7) yield where poor correlations were found (-1 to 1) (**Supplementary Table 5**).

**Table 3:** Statistical summary of the best linear unbiased estimators of key lentil traits.

Cluster	Yield mean (t/ha)	SD	No. of BLUEs	No. of locations	No. of genotypes	No. of environments
GYD	1.8	0.92	20,857	16	2,081	11
GYD-C1	2.59	0.79	5,114	8	1,610	8
GYD-C2	2.59	0.69	2,300	4	885	4
GYD-C3	1.3	0.74	6,183	6	1,880	9
GYD-C4	1.43	0.63	7,260	7	1,705	8
ABH	-0.32	0.58	899	1	823	5
ABN	0.33	1.11	864	1	617	5
BGM	5.82	1.48	3733	11	674	2
BOR	44.26	18.59	2626	1	1870	10
SAL	6.24	2.12	2533	1	1838	9
GWT	4.06	0.59	1298	4	635	3
SSI	4.46	0.25	1298	4	635	3
PRO	26.74	1.67	1298	4	635	3

### 3.3 Heritability and variance components

Broad sense heritabilities for GYD, the biotic and abiotic stress tolerance traits, as well as grain quality traits ranged from 0.47 to 0.83, whilst narrow sense heritabilities were lower, ranging from 0.24 to 0.66 (**Tables 4**, **5**).

**Table 4:** Broad-sense heritability (*H^2^*), narrow-sense heritability (*h^2^*), the additive genetic variance ( $\sigma_{a}^{2}$ ) and residual variance ( $\sigma_{e}^{2}$ ) for grain yield evaluation trial sites.

Clusters	*H^2^*	$\sigma_{a}^{2}$	$\sigma_{e}^{2}$	*h^2^*
GYD†	0.38	0.02	0.04	0.37
GYD-C1	0.43	0.02	0.05	0.26
GYD-C2	0.66	0.12	0.14	0.45
GYD-C3	0.28	0.01	0.02	0.33
GYD-C4	0.34	0.01	0.01	0.31

†Datasets in all clusters were combined, ignoring G×E interactions on GYD.

**Table 5:** Broad sense heritability (*H^2^*), narrow sense heritability (*h^2^*), the additive genetic variance ( $\sigma_{a}^{2}$ ) and residual variance ( $\sigma_{e}^{2}$ ) for measured biotic and abiotic stress tolerance and grain quality traits.

Traits	*H^2^*	$\sigma_{a}^{2}$	$\sigma_{e}^{2}$	*h^2^*
ABH	0.71	0.15	0.49	0.24
ABN	0.66	0.09	0.14	0.40
BOR	0.47	84.30	139.20	0.38
SAL	0.47	0.73	2.01	0.27
BGM	0.62	0.50	0.39	0.56
GWT	0.87	0.08	0.06	0.58
SSI	0.87	0.02	0.01	0.66
PRO	0.88	0.47	0.38	0.55

### 3.4 Genomic prediction accuracies for grain yield

**Figure 2:**
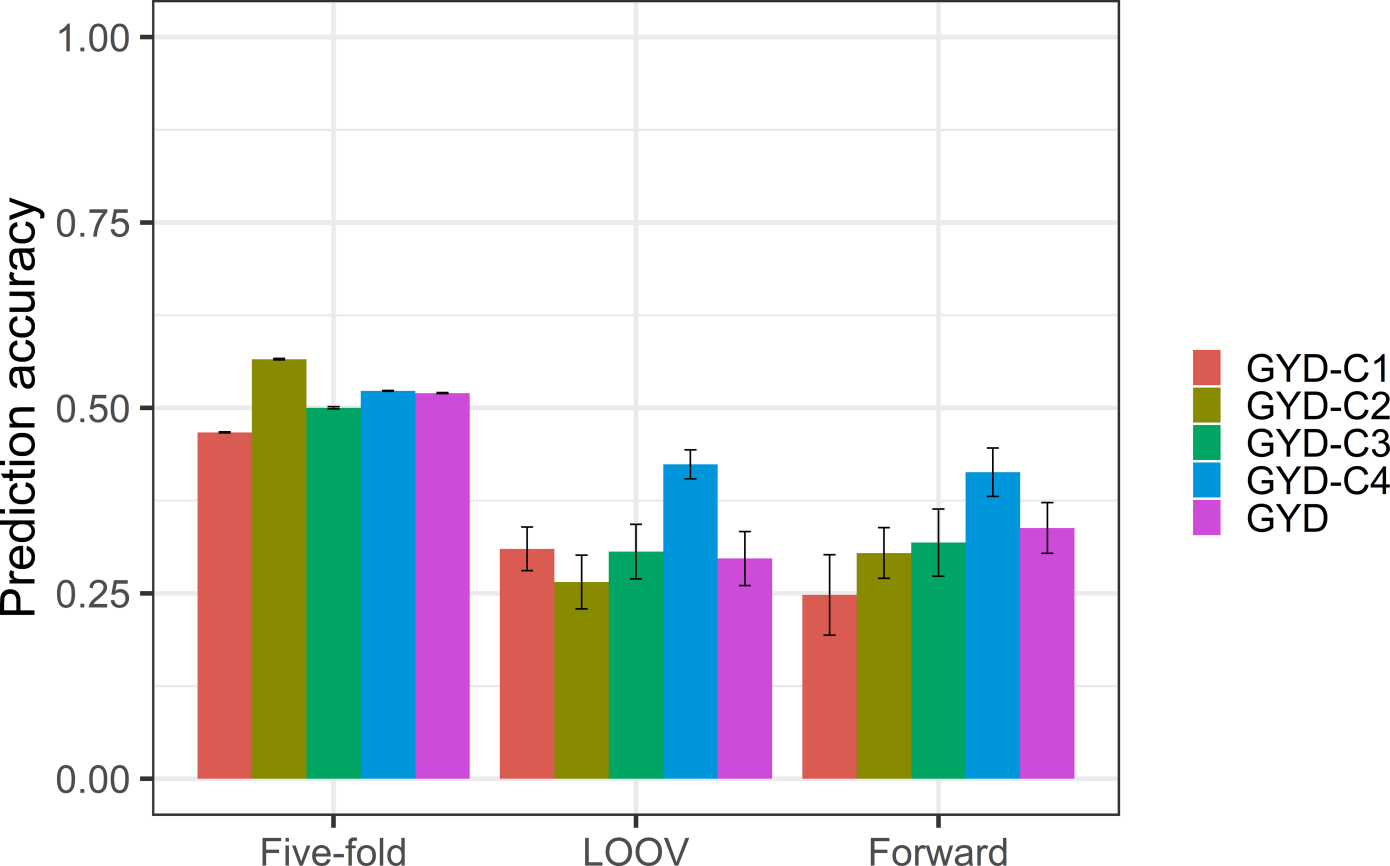
Prediction accuracies for grain yield clusters (standard error shown in error bar) achieved using three cross-validation methods: five-fold, LOOV and forward validation.

When comparing the three validation scenarios, random five-fold validation exhibited the highest prediction accuracy (0.47-0.57), where GYD-C2 showed the highest accuracy (0.57) and GYD-C1 the lowest (0.47) (**Figure 2**). The prediction accuracies for LOOV ranged from 0.27 to 0.42, with GYD-C4 (0.41) having the highest and GYD-C2 having the lowest (0.27) accuracy. The prediction accuracies for forward cross-validation ranged from 0.25 to 0.41, with GYD-C4 having the highest (0.41) and GYD-C1 having the lowest (0.25) accuracy. For yield without clustering (GYD), the accuracies ranged from 0.42-0.63 for all validation methods.

### 3.5 Genetic correlation between GYD clusters and prediction accuracies

**Figure 3:**
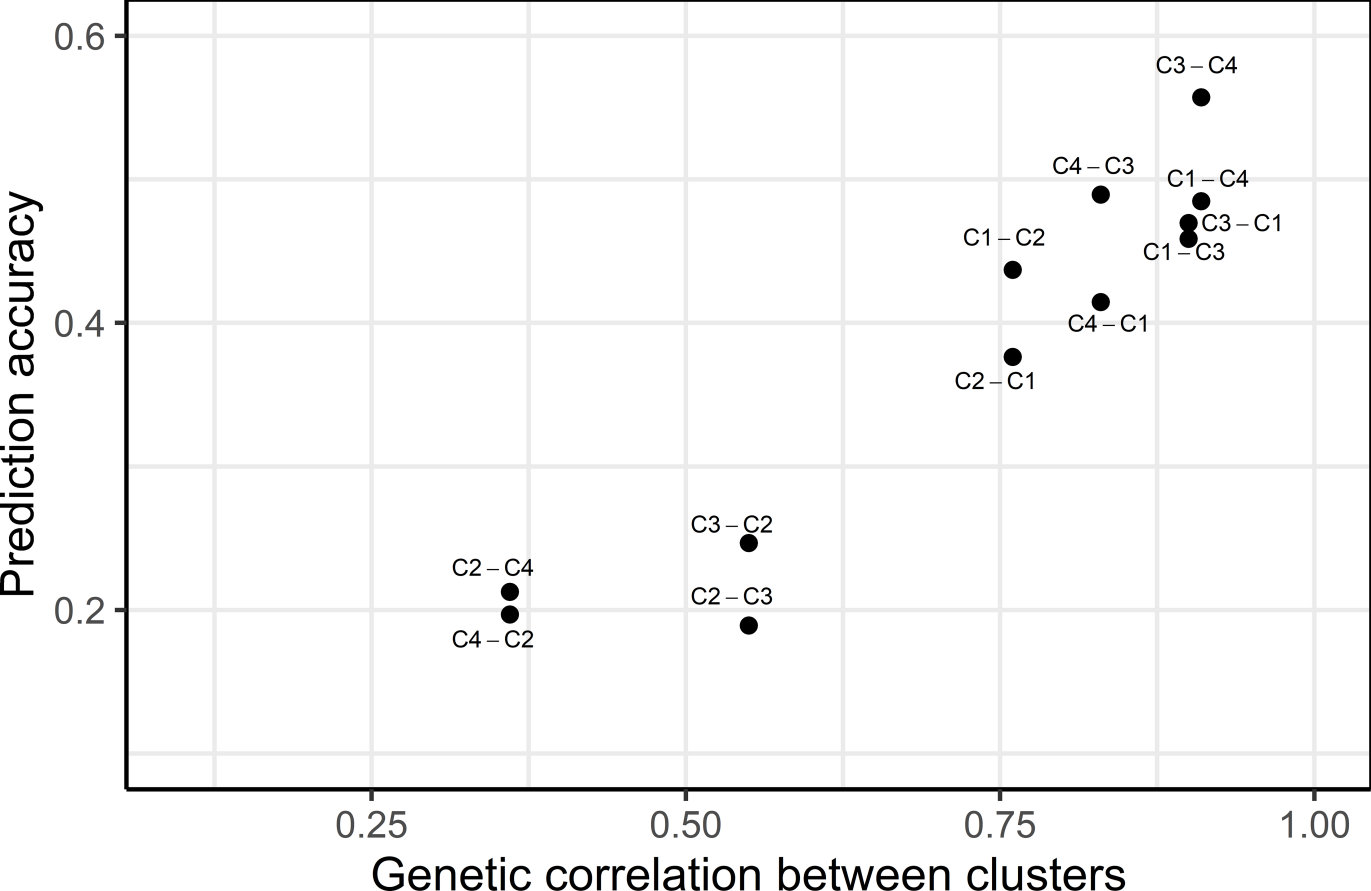
Relationship between prediction accuracy and genetic correlation between yield clusters. The text beside each dot point represents the training (left to hyphen) and the validation cluster (right to hyphen).

The prediction accuracy across clusters was found to be highly correlated to the genetic correlation between clusters, where higher genetic correlation led to higher prediction accuracy (**Figure 3**). Genetic correlations among GYD-C1, GYD-C3 and GYD-C4 were 0.83-1.00, implying a low level of G×E interaction among clusters. This led to a moderate prediction accuracy, ranging from 0.38 to 0.56. The correlation between GYD-C2 and the other clusters (GYD-C3, GYD-C4, and GYD-C1) was lower (0.36-0.76), indicating higher G×E interaction between GYD-C2 and other clusters. This resulted in weak to moderate prediction accuracies (0.19-0.44).

### 3.6 Prediction accuracies for abiotic and biotic stress tolerances and grain quality traits

**Figure 4:**
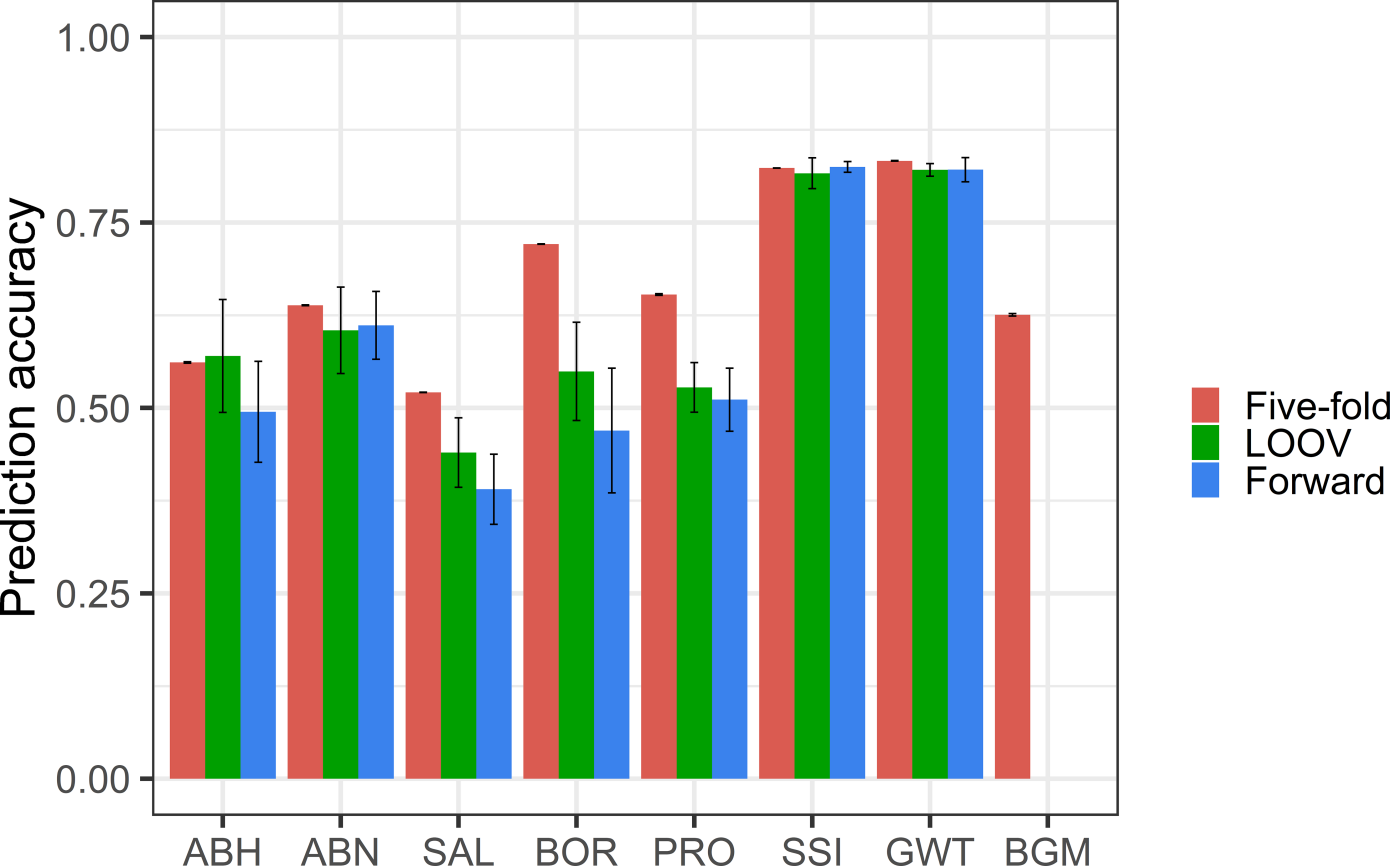
Prediction accuracies (standard error shown in error bar) from random five-fold, LOOV and forward cross-validation for abiotic and biotic stress tolerance and grain quality traits.

The prediction accuracy for ABH ranged from 0.45 to 0.57, with the highest achieved in LOOV and lowest in forward cross-validation (**Figure 4**). For ABN, similar prediction accuracy (0.60-0.64) was obtained with each validation method. For BGM, moderate prediction accuracy (0.63) was achieved using the five-fold method. The prediction accuracy could not be calculated for BGM using the LOOV and forward methods due to complete overlap of genotypes between the training and test sets.

For SAL, the prediction accuracy ranged from 0.39 to 0.52, with the highest achieved in random five-fold and lowest in forward cross-validation. For BOR, the prediction accuracy ranged from 0.47 to 0.72, with the highest achieved in random five-fold and lowest in forward cross-validation. The five-fold method had the highest prediction accuracies for both BOR (0.72) and SAL (0.52), while the forward prediction method had the lowest prediction accuracies, 0.47 and 0.39 respectively.

Prediction accuracies for grain quality traits GWT and SSI were 0.80 in all cross-validation methods. The prediction accuracy for PRO ranged from 0.51 to 0.65, with random five-fold achieving the highest and forward cross-validation having the lowest.

### 3.7 Comparison of GS to BLUP selection

The expected genetic gain per year from GS and BLUP selection when parents were selected at the stage 2, F_6_, F_2_ and F_1_ generation in the breeding cycle is shown in **Table 6**. In all scenarios, GS outperformed BLUP selection for all traits. When parents were chosen from stage 2, GS outperformed BLUP selection by 0.08 to 2.32-fold increase for all traits. Similarly, selecting parents from the F_6_ generation led to GS outperforming BLUP selection by 0.84 to 4.65 times. The additional expected genetic gain from GS over BLUP selection was 9.4 to 27.3-fold when selecting parents from the F_2_ generation, and 17.4 to 55.5-fold for selecting parents from the F_1_ generation.

**Table 6:** Expected genetic gain obtained from GS over BLUP selection. Genetic gain was calculated using the accuracy obtained from forward cross-validation on the parents from stage 2, F_6_, F_2_ and F_1._.

Trait	GS accuracy	BLUP accuracy	Stage 2 (fold)	F_6_ (fold)	F_2_ (fold)	F_1_ (fold)
GYD	0.34	0.26	0.22	1.08	9.4	19.8
BOR	0.39	0.34	0.08	0.84	8.2	17.4
SAL	0.47	0.30	0.47	1.50	11.5	24
ABH	0.49	0.14	2.33	4.65	27.3	55.5
ABN	0.61	0.31	0.86	2.16	14.8	30.6
BGM	0.63	0.45	0.31	1.22	10.1	21.2
PRO	0.51	0.22	1.19	2.72	17.6	36.2
SSI	0.82	0.54	0.44	1.44	11.2	23.4
GWT	0.82	0.57	0.36	1.31	10.5	22.1

## 4 Discussion

Over the years, lentil breeding in Australia has achieved significant success in enhancing grain yield through the utilization of conventional breeding methods and effective management practices. This continuous effort has resulted in an encouraging annual increase in the rate of genetic gain, averaging c. 1.2% over the past three decades (Sadras et al., 2021; Silva-Perez et al., 2022). However, to address increasing global demand for plant-based protein, it is crucial to explore new tools and technologies such as GS, which has the potential to further increase the rate of genetic gain, particularly in environments that are prone to abiotic and biotic stresses. GS enables more accurate and informed breeding selections, cost savings, and reduced breeding cycle times compared to traditional phenotype-based breeding approaches. Collectively, these advantages of GS lead to increased rates of genetic gain per unit time. Here, we report the implementation of GS for target traits in Agriculture Victoria’s national lentil breeding program. Ten years of historical phenotyping data (2010-2020) captured from advanced breeding stages under field and controlled environment conditions was used to train genomic prediction models for yield, biotic stress resistance, abiotic stress tolerance and seed quality traits. Various cross-validation methods achieved moderate to high prediction accuracies, which varied depending on trait complexity. Simple traits like boron tolerance had higher accuracies compared to complex traits like GYD. Despite this, GYD still had moderate prediction accuracies, making GS a preferred method for lentil breeders. The observed accuracies are comparable to previous studies in pulse crops (Li et al., 2018; Annicchiarico et al., 2019; Keller et al., 2020; Bari et al., 2021; Diaz et al., 2021).

In commercial breeding programs, addressing the G × E interaction is crucial to achieve yield stability, as it results in varying genotype performance across different environments. Including G × E components in GS models has been reported to improve prediction accuracies. To assess the G x E levels in the current dataset, pairwise genetic correlations were calculated among various GYD trials conducted from 2010 to 2020 (data not shown). The results revealed relatively low correlations, which could be attributed to the limited number of overlapping entries between these trials, resulting in low connectedness. As an alternative approach, environmental variables were utilized to cluster the GYD data into four groups, primarily based on total rainfall and evaporation, which accounted for most of the variation. Clustering of mega-environments into groups based on environmental variables has been reported in barley (Heslot et al., 2013; Lin et al., 2021) and wheat (Dawson et al., 2013). Genetic correlations were high among GYD-C1, GYD-C3, and GYD-C4 (exceeding 0.80), but lower with GYD-C2 (0.36 to 0.76). Predicting GYD-C2 using GYD-C3 and GYD-C4, and vice versa, resulted in lower accuracies (0.19-0.25). This could be due to GYD-C2 having only 14 trials over four years (2010-2014), while GYD-C3 and GYD-C4 spanned 33-47 trials over ten years (2010-2020), leading to lower connectedness between GYD-C2 and the other clusters. This would also mean the training population of GYD-C2 would be genetically more distant (less related) when compared to GYD-C3 and GYD-C4. Despite this, GYD-C2 exhibited higher heritability estimates, possibly due to higher relatedness within its training population. Overall, clustering based on environmental variables did not significantly improve GYD predictions compared to non-clustering methods, as environmental variables are just one factor influencing plant performance.

In Australia, lentils are a high-value cash crop, with more than 95% of the harvest exported to the global market. Consequently, grain quality traits such as seed size, protein content, and seed colour are priority breeding targets. However, breeding for quality traits poses further challenges because of the commonly observed negative correlation between seed protein content and grain yield in pulse crops such as chickpea, common bean and pigeon pea (Saxena et al., 1987; Gaur et al., 2016; Keller et al., 2020). When traits are antagonistically connected, it adds another layer of complexity for the simultaneous improvement of multiple traits. To address this complexity, different strategies can be implemented in a breeding program. One strategy is to apply selection at an early stage of the breeding program, when a larger number of early generation progenies are available and stronger selection can be applied. With larger progeny numbers, the likelihood of identifying individuals with favourable alleles for both grain yield and protein content is increased. A wheat GS study found a positive association between grain yield and protein content in some progeny groups, despite the fact that the corresponding parents were negatively correlated (Yao et al., 2018). However, while this may result in short-term success, it will result in the loss of the highest yielding genotypes in the breeding program due to segregation. Another option is the use of a selection index, where multiple traits of importance are indexed simultaneously (weighted selection index, culling and tandem selection, index selection or independent culling) (Batista et al., 2021; Covarrubias-Pazaran et al., 2021). A weighted selection index is calculated by assigning a certain weight to each trait based on their relative economic values. In the absence of economic weights, breeders can define targeted genetic gain based on long-term breeding goals. This allows the balance between favorable genetic gain and targeted genetic gain to be optimized to overcome the problem of targeted gain in which selection for one trait may lead to unfavorable changes in other traits due to correlations between traits. Breeders may also aim to develop product classes (also known as target product profiles) to maintain balance in the breeding program and meet export demands for different markets. For instance, Agriculture Victoria’s national lentil breeding program is targeting different export markets who have demand for small and large seed size lentils. To clearly define these product profiles, we are implementing weighted selection indices to underpin parental selections for hybridization programs. Based on these decisions, crossing designs, cross evaluation, and selection tasks are carried out for each individual product profile. This means that selection decisions are made at various breeding stages including intercrosses, bulk-up, preliminary yield trials, Stage 1 trials, and Stage 2 trials (evaluation of advanced breeding lines).

As grain yield and quality in lentils can be significantly impacted by biotic and abiotic stresses, (Araujo et al., 2015; Li et al., 2015; Scheelbeek et al., 2018; Dutta et al., 2022), it is imperative to target breeding for disease resistance and abiotic stress tolerance. Both AB and BGM affect lentil production in Australia. For biosecurity reasons, glasshouse assays are used to screen genotypic responses to AB resistance. The prediction accuracy (0.45-0.64) for AB resistance observed in this study was moderately high indicating that GS could replace some of the phenotypic screens as a selection tool for this trait. BGM assays, on the other hand, rely on field endemics that occur under high rainfall conditions. Given the pathogen’s complexity and the low frequency of high rainfall seasons across lentil growing regions in Australia, breeders have relied on opportunistic field scores. In this study, moderately high prediction accuracy (0.62) was obtained using data captured from two seasons (2013 and 2016), which is sufficient to enable GS to be applied for this trait in the breeding program. Similarly, moderate to high prediction accuracies observed for both boron and salt tolerance (0.39-0.72) (**Figure 4**) demonstrated the utility of GS for developing abiotic stress tolerant lentil varieties. In conclusion, incorporating these GS results into a breeding strategy will help to accelerate the development of lentil cultivars with quantitative disease resistance and abiotic stress tolerance.

Early parent selection in GS is critical for shortening the breeding cycle and lowering breeding costs. When GS is used in the breeding program, it is expected to outperform phenotypic selection in terms of accuracy and genetic gain per unit time (Matei et al., 2018; Li et al., 2022). In this study, GS was shown to provide significant genetic gain over BLUP based selection when parents were selected from stage 2, F6, F2 and F1 (**Table 6**). Noteworthy was that prediction accuracy was reduced when parents were selected from earlier generations. This was likely caused by the training population only being updated with phenotyped Stage 2 and 3 materials. Historically, updating the parents for selection of the next breeding cycle could take up to 8 years (F8 or stage 2). However, with the availability of genomic prediction, parental selection can be carried out earlier in the breeding program. This is beneficial for shortening the breeding cycle and cycling back superior individuals from crosses as parents into subsequent hybridization cycles. (Li et al., 2022) suggested that using phenotyped F_2_ families to update the prediction model each breeding cycle improved prediction accuracy. Another point worth mentioning is that increased genetic gain per unit of time resulting from the selection of earlier generation parents will result in the rapid loss of genetic diversity (inbreeding). To mitigate this, breeders can use different strategies including introducing exotic germplasm into crossing blocks and using computational algorithms that help chose optimal parents while maintaining diversity (Lin et al., 2017). Li et al. (2022) developed a strategy to maximise genetic gain while preserving genetic diversity by restricting the co-ancestry of selected parents and the number of alleles fixed. Similarly, (Gorjanc et al., 2018) developed a method for comparing optimal cross-selection and truncation selection, and optimal cross-selection to increase long-term genetic gain while preserving diversity. In conclusion, this study demonstrates the potential of GS for making informed selections in breeding programs and for cycling parents back from earlier stages to accelerate the development of high yielding, biotic and abiotic stress tolerant, lentil varieties with superior grain quality.

## Data availability statement

The minimum data set required to undertake the analysis included in the manuscript is provided in Supplementary files. We note that both the sample and marker names in the genotyping data file have been de-identified due to the data originating from a commercial breeding program. For any additional inquiries, please contact the corresponding author.

## Author contributions

AG: Formal Analysis, Investigation, Methodology, Software, Visualization, Writing – original draft. YL: Conceptualization, Methodology, Software, Supervision, Visualization, Writing – review & editing. AS: Data curation, Methodology, Validation, Writing – review & editing. SS: Data curation, Formal Analysis, Methodology, Writing – review & editing. HV-K: Formal Analysis, Investigation, Methodology, Software, Visualization, Writing – review & editing. MH: Funding acquisition, Project administration, Resources, Writing – review & editing. GR: Funding acquisition, Methodology, Project administration, Resources, Supervision, Validation, Writing – review & editing. SK: Conceptualization, Funding acquisition, Investigation, Methodology, Project administration, Resources, Supervision, Writing – review & editing.
