# Supplementary material for: Genomic selection for target traits in the Australian lentil breeding program
Source: Front Plant Sci. 2024 Jan 3;14:1284781. doi: 10.3389/fpls.2023.1284781 (PMC10791954; doi:10.3389/fpls.2023.1284781)
Supplement: Supplementary file 1 [file DataSheet_1.zip › Table 1 - 2023-12-07T123811.187.DOCX]

Supplementary Table 1: Geographic locations of the experimental sites

| Experimental site | State |
| --- | --- |
| BEULAH | VIC |
| CULGOA | VIC |
| HOPETOUN | VIC |
| HORSHAM | VIC |
| CURYO | VIC |
| MALLALA | SA |
| MINYIP | VIC |
| MELTON | SA |
| RUPANYUP | VIC |
| KINGSFORD | NSW |
| SALMONGUMS | WA |
| WAGGAWAGGA | NSW |
| GRASSPATCH | WA |
| SCADDEN | WA |
| KADINA | SA |
| WILLAMULKA | SA |
