# Supplementary material for: Genomic selection for target traits in the Australian lentil breeding program
Source: Front Plant Sci. 2024 Jan 3;14:1284781. doi: 10.3389/fpls.2023.1284781 (PMC10791954; doi:10.3389/fpls.2023.1284781)
Supplement: Supplementary file 1 [file DataSheet_1.zip › Table 3 (59).DOCX]

Supplementary Table 3: Details of Ascochyta lentis isolates

| Year of testing | Isolate Name | Isolate type |
| --- | --- | --- |
| 2016 | F15198 | Nipper-virulent |
| 2016 | FT14125 | "Horsham isolate" = Hurricane virulent |
| 2017 | F15198 | Nipper-virulent |
| 2017 | F16299-2 | Hurricane-virulent |
| 2018 | F17083 | Hurricane-virulent |
| 2019 | FT18086 | Hurricane-virulent |
| 2018 | FT17197-2 | Nipper-virulent |
| 2019 | FT18087 | Nipper-virulent |
| 2020 | FT18087 | Nipper-virulent |
| 2020 | FT18042-1 | Hurricane-virulent |
