# Supplementary figures and images for: Genomic selection for target traits in the Australian lentil breeding program
Source: Front Plant Sci. 2024 Jan 3;14:1284781. doi: 10.3389/fpls.2023.1284781 (PMC10791954; doi:10.3389/fpls.2023.1284781)

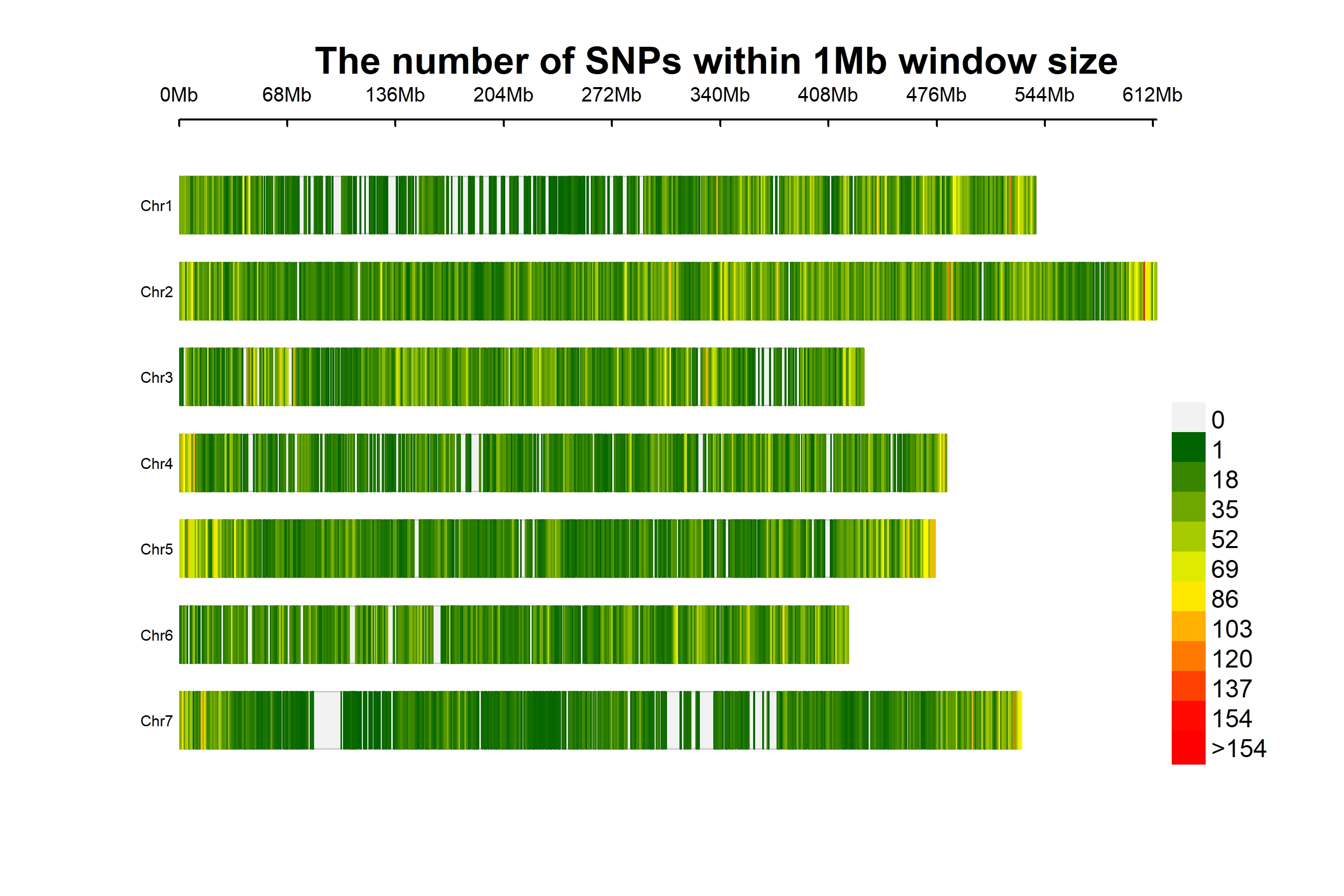

Supplement: Supplementary file 1 [file DataSheet_1.zip › Image 1 (5).TIFF]
